# Escitalopram oxalate induces apoptosis in U‐87MG cells and autophagy in GBM8401 cells

**DOI:** 10.1111/jcmm.13372

**Published:** 2017-11-03

**Authors:** Vincent Chin‐Hung Chen, Yi‐Hsien Hsieh, Li‐Jeng Chen, Tsai‐Ching Hsu, Bor‐Show Tzang

**Affiliations:** ^1^ Department of Psychiatry Chang Gung University Taoyuan Taiwan; ^2^ Department of Psychiatry Chiayi Chang Gung Memorial Hospital Chiayi Taiwan; ^3^ Clinical Laboratory Chung Shan Medical University Hospital Taichung Taiwan; ^4^ Institute of Biochemistry Microbiology and Immunology Chung Shan Medical University Taichung Taiwan; ^5^ Department of Biochemistry School of Medicine Chung Shan Medical University Taichung Taiwan; ^6^ Immunology Research Center Chung Shan Medical University Taichung Taiwan

**Keywords:** glioblastoma multiforme, brain cancer, selective serotonin reuptake inhibitor, escitalopram oxalate, U‐87MG

## Abstract

Glioblastoma multiforme (GBM) is recognized as a most aggressive brain cancer with the worst prognosis and survival time. Owing to the anatomic location of gliomas, surgically removing the tumour is very difficult and avoiding damage to vital brain regions during radiotherapy is impossible. Therefore, therapeutic strategies for malignant glioma must urgently be improved. Recent studies have demonstrated that selective serotonin reuptake inhibitors (SSRIs) have cytotoxic effect on certain cancers. Considering as a more superior SSRI, escitalopram oxalate exhibits favourable tolerability and causes generally mild and temporary adverse events. However, limited information is revealed about the influence of escitalopram oxalate on GBM. Therefore, an attempt was made herein to explore the effects of escitalopram oxalate on GBM. The experimental results revealed that escitalopram oxalate significantly inhibits the proliferation and invasive ability of U‐87MG cells and significantly reduced the expressions of cell cycle inhibitors such as Skp2, P57, P21 and P27. Notably, escitalopram oxalate also induced significant apoptotic cascades in U‐87MG cells and autophagy in GBM8401 cells. An animal study indicated that escitalopram oxalate inhibits the proliferation of xenografted glioblastoma in BALB/c nude mice. These findings implied that escitalopram oxalate may have potential in treatment of glioblastomas.

## Introduction

Malignant gliomas are known as the most common brain tumours. Gliomas are classified into four grades (Grades I–IV) according to histology and prognosis 1. GBM is a grade IV astrocytoma and the most aggressive brain cancer with the execrable prognosis and survival rate 2, 3. The ordinary consideration for GBM treatment is surgical resection in combination with radiotherapy and chemotherapy. However, the anatomic location of gliomas makes surgically removing the tumour very difficult and avoiding damage to vital brain regions during radiotherapy is impossible. Even though temozolomide chemotherapy and radiotherapy are combined with surgical resection, the average survival time in patients with grade IV glioma is nearly one and half year 4. Thus, the alternative strategy for treatment of malignant glioma must be earnestly improved.

The cancer patients suffering from depressive disorders are commonly prescribed with antidepressants. Notably, recent retrospective studies have indicated that treatment with tricyclic antidepressants (TCAs) has the negatively association with the risk of gliomas and that SSRI reduce the proliferation and induce cytotoxic effect on certain cancers 5, probably because it can pass through the BBB and have a direct pharmaceutical effect in certain regions of the brain 6, 7. A recent study showed that fluoxetine, a frequently used SSRI, may be selectively toxic to gliomas by generating an overloading of mitochondrial calcium ions (Ca^2+^), triggering apoptosis 8. These findings indicate a possibility that antidepressants have a better effect on brain cancers treatment than chemotherapeutic agents.

Escitalopram oxalate, also known as Cipralex^®^ and Lexapro^®^, is a selective serotonin reuptake inhibitor (SSRI) that is frequently used to treat major depressive disorder (MDD) and anxiety disorder. According to a meta‐analysis and a pooled analysis, escitalopram oxalate has advantages over other antidepressants 9. Various studies have demonstrated escitalopram oxalate as a more superior antidepressant than placebo, other SSRIs such as citalopram, paroxetine, fluoxetine and sertraline, and serotonin‐noradrenaline reuptake inhibitors such as duloxetine and sustained‐release venlafaxine. Escitalopram oxalate also exhibits favourable tolerability and causes generally mild and temporary adverse events 9, 10, 11, 12. However, limited information is known about the effects and the possible mechanism of escitalopram oxalate on GMB.

## Materials and methods

### Cell culture and selective serotonin reuptake inhibitor (SSRI)

Human brain astrocyte cells (HBA) and human malignant gliomas cell lines, GBM8401, U‐87MG and Hs683, were purchased from ScienCell (CA, USA) and ATCC (Rockville, MD, USA), respectively. Both cells were cultured in EMEM supplemented with 2 mM glutamine, 1% non‐essential amino acids (NEAA), 1 mM sodium pyruvate (NaP) and 10% foetal bovine serum (Gibco, Life Technologies Co, Grand Island, NY, USA). The selective serotonin reuptake inhibitor (SSRI), escitalopram (Lexapro, St. Louis, MO, USA), was provided by Chiayi Chang Gung Memorial Hospital.

### Flow cytometric analysis

A total number of 1 × 10^6^ cells were seeded in culture plates at per 100 mm^2^ and cultured with serum‐free medium for 24 hrs at 37°C in a 5% CO_2_ incubator. Cells were then incubated with different concentrations of escitalopram oxalate for another 24 hrs. After incubation, cells were harvested, washed with PBS and fixed with 70% alcohol for 16 hrs at 4°C. The cells were then washed with PBS and transferred into 12 × 75‐mm tubes. A total of 10 μl of propidium iodide (PI) staining solution were added, gently mixed and incubated in ice bath at dark. After filtration through a 40‐μm nylon screen, the stained cells were analysed with a FACS Calibur analyzer (Becton Dickinson, Bedford, MA, USA).

### MTT assay

A total of 5 × 10^3^ cells were seeded in each well of a 96‐well plate and cultured in EMEM containing 10% FBS overnight. The culture medium was replaced with fresh medium containing different concentrations of escitalopram oxalate for 1 or 2 day, respectively. After incubated with 0.2 ml MTT for another 2 hrs, a 0.2 ml DMSO was then added to each well of the plate to dissolve the crystal. The absorbance of the supernatant was measured at 570 nm with an ELISA reader, and the cell survival rate was presented as the ratio of the absorbance of the sample treatment relative to the control. The inhibitory concentration (IC) 50 rate of escitalopram oxalate on U‐87MG cells was calculated as described elsewhere 13.

### Wound healing assay

Cell motility was detected using a wound healing assay as described in a previous study 14. The cells were incubated with EMEM medium containing 10% FBS at in 37°C incubator. Briefly, the cell monolayer was scratched a line with a plastic scriber. After incubation for 16 hrs, the distance moved by the advancing cells was measured. Photographs were taken under a phase‐contrast microscope (at ×400 magnification).

### Cell migration assay

A cell invasion assay was conducted in 24‐well Matrigel™ Invasion inserts (8 μm pore size; BD Biosciences, Franklin Lakes, NJ, USA) to measure the effects of escitalopram oxalate. Briefly, 2 × 10^4^ cells in serum‐free DMEM that contained various concentrations of escitalopram oxalate were loaded into the upper chamber and DMEM that contained 10% foetal bovine serum was added to the lower chamber. After 2 days of incubation, the cells on the upper surface of the membranes were removed using a cotton swab. Migrated cells on the lower surface of the membrane were fixed with 10% neutral‐buffered formalin and stained with 0.5% crystal violet for 15 min. Three independent assessors were then performed by counting the cells in five randomly selected microscopic fields at 200× magnification per filter.

### Immunoblotting

Immunoblotting was performed as described elsewhere 15. Briefly, antibodies against Skp2, p57, p21, p27, Bax, cytochrome c, Apaf‐1, caspase‐3, caspase‐9, PARP (Santa Cruz Biotechnology, Santa Cruz, CA, USA), Atg‐3 (R&D Systems Inc., Minneapolis, MN, USA), LC3, Beclin‐1, Atg‐5, Atg‐7, Atg‐9, p62 (Abcam Ltd., Cambridge, UK) and β‐actin (Upstates, Charlottesville, VA, USA) were diluted in PBS with 2.5% BSA and incubated for 3 hrs with gentle agitation at room temperature. After the incubation, secondary antibody conjugated with horseradish peroxidase (HRP) was added to react for another 1 hr. Immobilion Western HRP Chemiluminescent Substrate (Millipore, Temecula, USA) was then used to detect the antigen–antibody complexes. The blots were scanned and quantified by densitometry (Appraise, Beckman‐Coulter, Brea, CA, USA).

### Immunofluorescence staining

GBM8401 cells cultured on coverslips were fixed in 4% paraformaldehyde. After permeabilized with 0.3% Triton X‐100 for 5 min., the coverslips were then incubated in blocking solution at room temperature, followed by hybridizing with anti‐LC3‐II antibodies (Sigma‐Aldrich, St. Louis, MO, USA). Next, the cells were incubated with Alexa Fluor^®^ conjugated secondary antibodies (Abcam Ltd.) for 1 hr and washed with PBS. The coverslips were mounted in ProLong™ Gold Antifade Mountant with DAPI (Thermo Fisher Scientific Inc., Wilmington, MA, USA) for 5 min. and observed under a ZEISS AXioskop2 fluorescence microscope **(**Carl Zeiss Microscopy; Thornwood, NY, USA).

### Animals and tumour xenografts

Twenty‐four male athymic nude mice (BALB/c nude mice) at 5‐week old were purchased from the National Center for Experimental Animals (National Science Council, Taiwan). All mice were maintained in a specific pathogen‐free facility at 22°C with a 12‐hr light/12‐hr dark cycle. The animals were given to adapt to the environment for 1 week after their arrival before the experiment began. All protocols were approved by the Institutional Animal Care and Use Committee of Chung Shan Medical University, Taichung, Taiwan (IACUC approval number: 1584). The principles of laboratory animal care (NIH publications) were followed. In xenograft experiments, U‐87 MG cells (5 × 10^6^ cells in 100 μl PBS) were subcutaneously injected into the flank of the mice at an age of 6 weeks. 7 days after the injection of U‐87MG cells, the mice were randomly assigned to three groups, which were the control group, the low‐dose group and the high‐dose group. The low‐dose and high‐dose groups were administered daily 2.5 mg/kg or 12.5 mg/kg escitalopram oxalate by oral gavage. The control group was administered daily PBS by oral gavage. Tumour diameters were measured weekly, and tumour volumes were calculated. After 21 days of treatment, the mice were killed and the tumours were harvested, weighed and fixed in 4% paraformaldehyde for immunohistochemical analysis.

### Immunohistochemical staining

Tumour tissue sections were performed as described elsewhere 16. The tissue samples were excised and soaked in formalin and covered with wax. Slides were prepared by deparaffinization and dehydration. The slides were then subjected to immunohistochemical stating with a rabbit antiproliferating cell nuclear antigen (PCNA) antibody (Santa Cruz Biotechnology) and revealed using a polymer detection system kit (Leica Biosystems, Mount Waverley, VIC, Melbourne, Australia). A Zeiss Axiophot microscope was used to take photomicrographs of the sections. At least six tumour sections from three different mice were analysed.

### Statistical analysis

GraphPad Prism 5 software (GraphPad Software, La Jolla, CA, USA) by one‐way analysis of variance (one‐way anova) was adopted to perform the statistical analyses. Tukey's multiple comparisons test was also performed to determine the significance. Data were represented as mean ± S.E.M. Three repeated experiments were performed. A *P* value < 0.05 was considered statistically significant.

## Results

### Escitalopram oxalate inhibits the proliferation and invasive ability of U‐87MG cells

**Figure 1:**
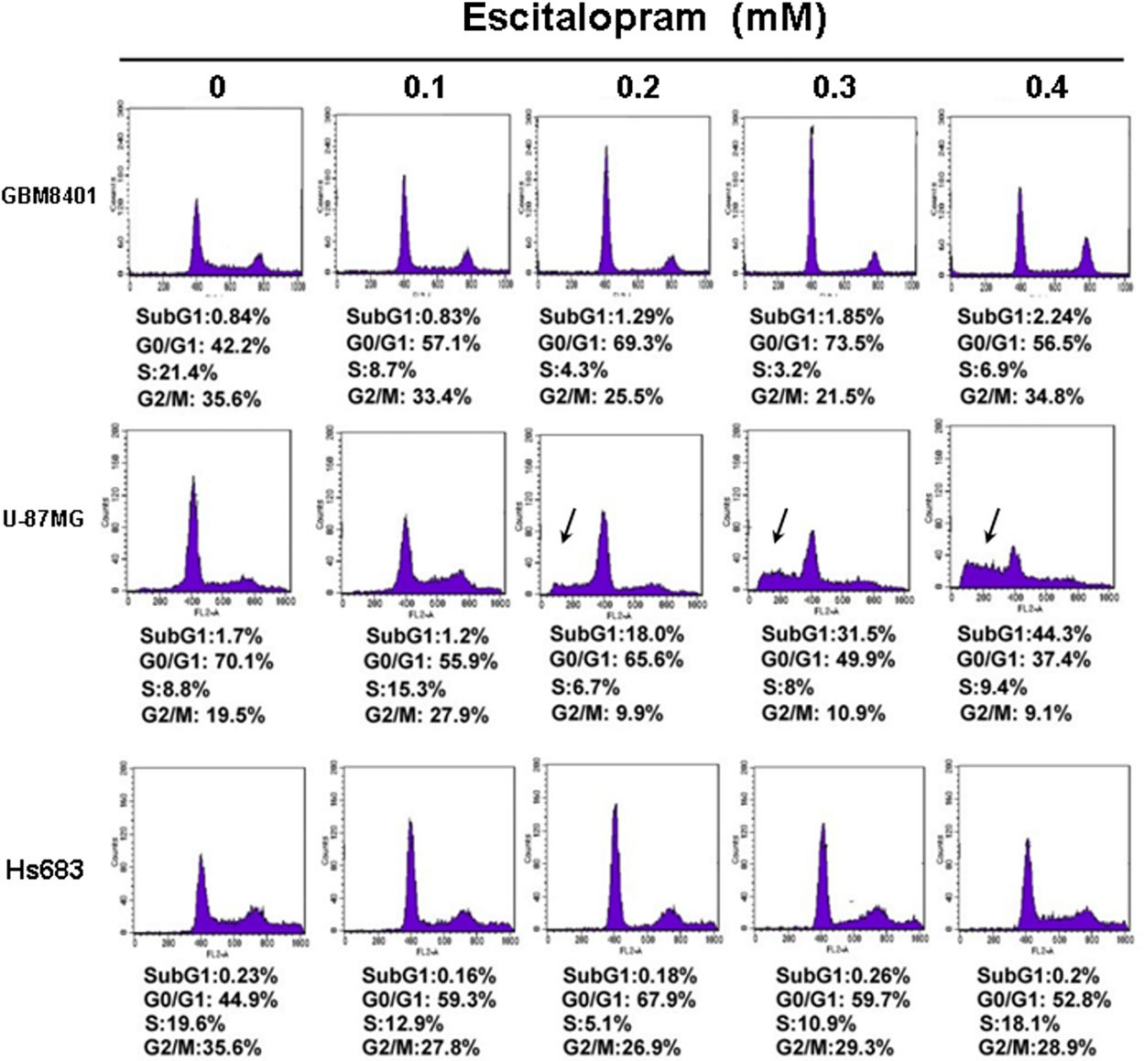
Representative results of flow cytometry in GBM8401, U‐87MG and Hs683 cells in the presence of escitalopram oxalate for 24 hrs. Arrow indicated the sub‐G1 proportion. Similar results were observed in three repeated experiments.

**Figure 2:**
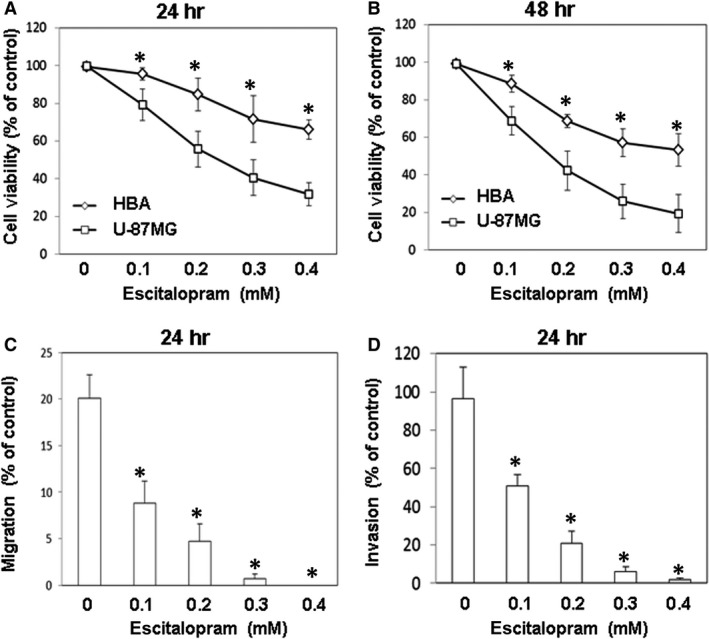
The effects of escitalopram oxalate on the viability, motility and invasive abilities of U‐87MG and human brain astrocyte (HBA) cells. Relative cell survival of U‐87MG and HBA was detected after treatment with different concentrations of escitalopram oxalate for (**A**) 24 and (**B**) 48 hrs. (**C**) Wound‐healing assay and (**D**) transwell migration assay were performed in U‐87MG cells treated with different concentrations of escitalopram oxalate for 24 hrs. Similar results were observed in three repeated experiments, and * indicates the significant difference, *P* < 0.05.

To examine the effects of escitalopram oxalate on glioblastoma, GBM8401, U‐87MG and Hs683 cells were analysed with flow cytometry in the presence of different concentrations of escitalopram oxalate for 24 hrs (Fig. 1). Notably, apparently increased sub‐G1 population was detected in U‐87MG cells in the presence of escitalopram oxalate at the concentrations of 0.2, 0.3 and 0.4 mM but not in other cell lines (Fig. 1). To further evaluate the effects of escitalopram oxalate on human glioblastoma astrocytoma, U‐87MG and human brain astrocyte (HBA) cells were treated with various doses of escitalopram oxalate and then analysed using MTT, wound healing and trans‐well migration assays. Treatment with 0.1, 0.2, 0.3 or 0.4 mM escitalopram oxalate for 24 hrs significantly reduced the survival rate of U‐87MG cells, relative to that of HBA cells (Fig. 2A). Similar results were observed for U‐87MG cells after 48‐hr treatment with escitalopram oxalate (Fig. 2B). The IC_50_ of escitalopram oxalate on U‐87MG cells was 0.247 and 0.169 mM at the time‐points of 24 and 48 hrs, respectively. The motility and invasive ability of U‐87MG cells were further examined. Significantly reduced migration was observed in U‐87MG cells that were treated with 0.1, 0.2, 0.3 or 0.4 mM escitalopram oxalate, relative to the cells in the control group (Fig. 2C). As revealed by the transwell invasion chamber assay, the number of invaded U‐87MG cells that were treated with 0.1, 0.2, 0.3 or 0.4 mM escitalopram oxalate was less than the number of invaded cells in the control group (Fig. 2D).

### Escitalopram oxalate induces the expression of cell cycle inhibitors in U‐87MG cells

**Figure 3:**
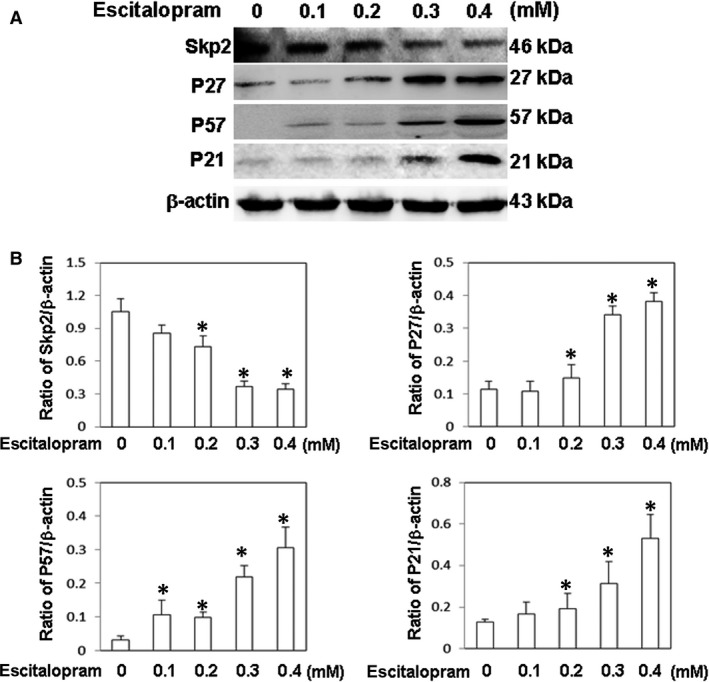
Expressions of cell cycle‐related proteins. (**A**) The expressions of Skp2, P27, P57 and p21 proteins in U‐87MG cells that were treated with different concentrations of escitalopram oxalate. (**B**) Bars represent the relative protein quantification on the basis of β‐actin. Similar results were observed in three repeated experiments, and * indicates the significant difference, *P* < 0.05.

To elucidate the effect of escitalopram oxalate on cell cycle‐related molecules, the expressions of various cell cycle inhibitors such as p57, p21 and p27 proteins were evaluated using immunoblotting. The amounts of p57, p21 and p27 proteins in U‐87MG cells significantly increased with the concentration of escitalopram oxalate (Fig. 3A), whereas the concentration of Skp2, a specific inhibitor of protein p27, in U‐87MG cells significantly declined (Fig. 3A). The lower panel of Figure 3B presents quantities of p57, p21, p27 and Skp2 relative to that of β‐actin.

### Escitalopram oxalate induces the expression of apoptotic proteins in U‐87MG cells

**Figure 4:**
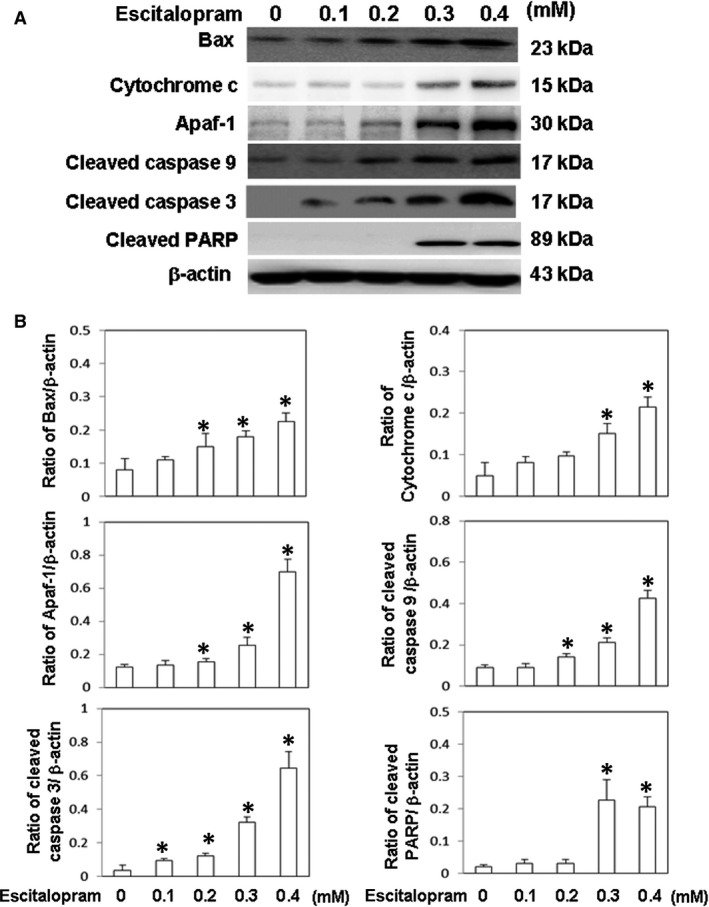
Expressions of apoptotic‐related proteins. (**A**) The expressions of Bax, cytochrome c, Apaf‐1, cleaved caspase 9, cleaved caspase 3 and cleaved PARP proteins in U‐87MG cells that were treated with different concentrations of escitalopram oxalate. (**B**) Bars represent the relative protein quantification on the basis of β‐actin. Similar results were observed in three repeated experiments, and * indicates the significant difference, *P* < 0.05.

To determine whether escitalopram oxalate induces cell death in U‐87MG cells, immunoblotting was performed to detect the expressions of various apoptotic proteins, including Bax, cytochrome c, Apaf‐1, caspase‐3, caspase‐9 and PARP. The expressions of Bax, cytochrome c, Apaf‐1, caspase‐3, caspase‐9 and PARP were significantly increased in U‐87MG cells with the concentration of escitalopram oxalate (Fig. 4A). The lower panel in Figure 4B presents quantitative results concerning the amounts of Bax, cytochrome c, Apaf‐1, caspase‐3, caspase‐9 and PARP relative to that of β‐actin.

### Escitalopram oxalate induces autophagy in GBM8401 cells

**Figure 5:**
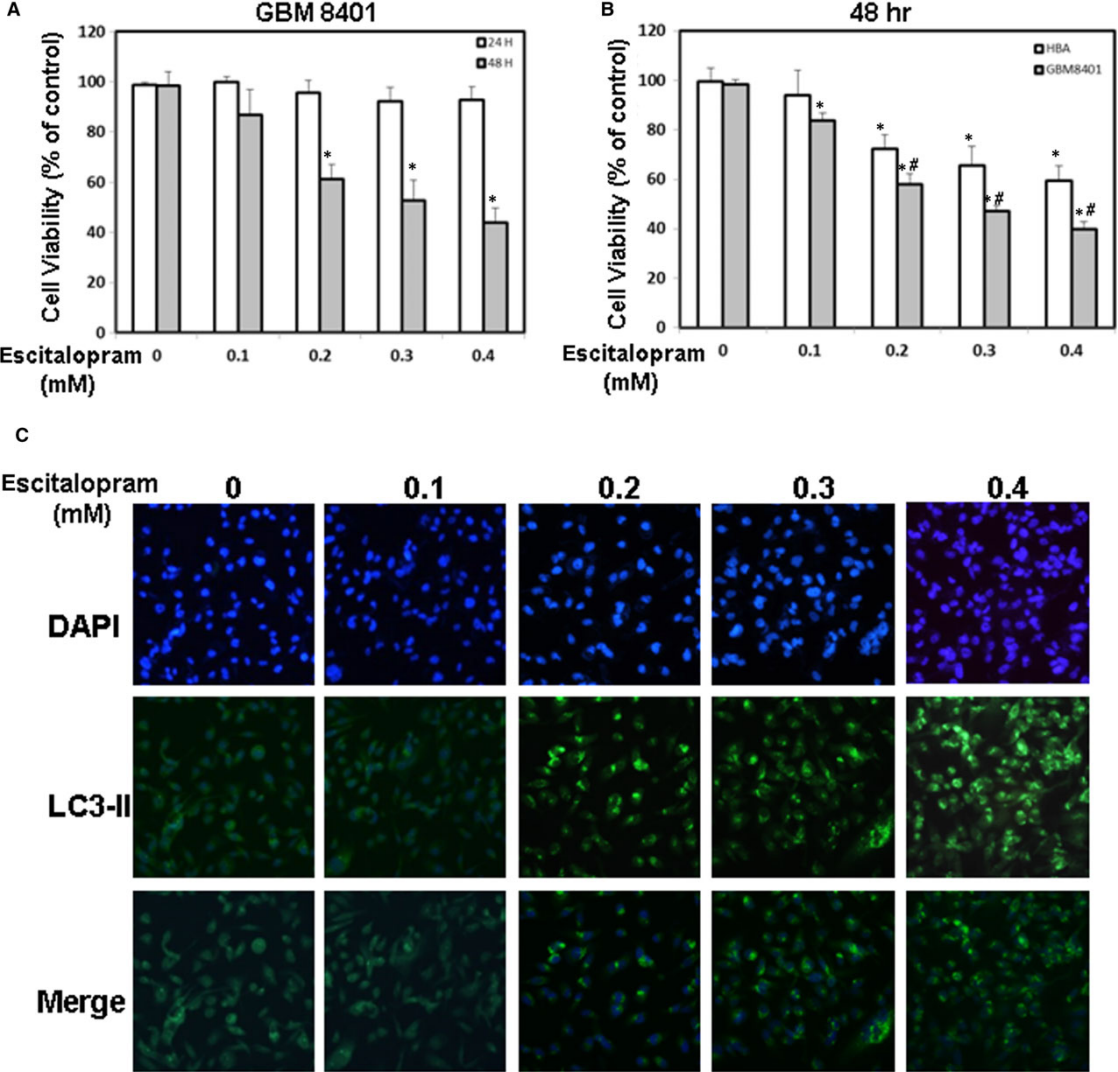
The effects of escitalopram oxalate on the viability of GBM8401 cells. (**A**) The cell survival ratio of GBM8401 cells was detected after treatment with different concentrations of escitalopram oxalate for 24 and 48 hrs. (**B**) Relative cell survival of GBM8401 and HBA was detected after treatment with different concentrations of escitalopram oxalate for 48 hrs. (**C**) The autophagy was detected by immunofluorescence staining. GBM8401 cells were treated with different concentrations of escitalopram oxalate for 48 hrs and reacted with DAPI (upper panel) and antibodies against LC3‐II (middle panel). Lower panel indicates the merged images of DAPI and LC3‐II. Similar results were observed in three repeated experiments. *and ^#^indicate the significant difference, *P* < 0.05, as compared to control (0 mM) and HBA, respectively.

**Figure 6:**
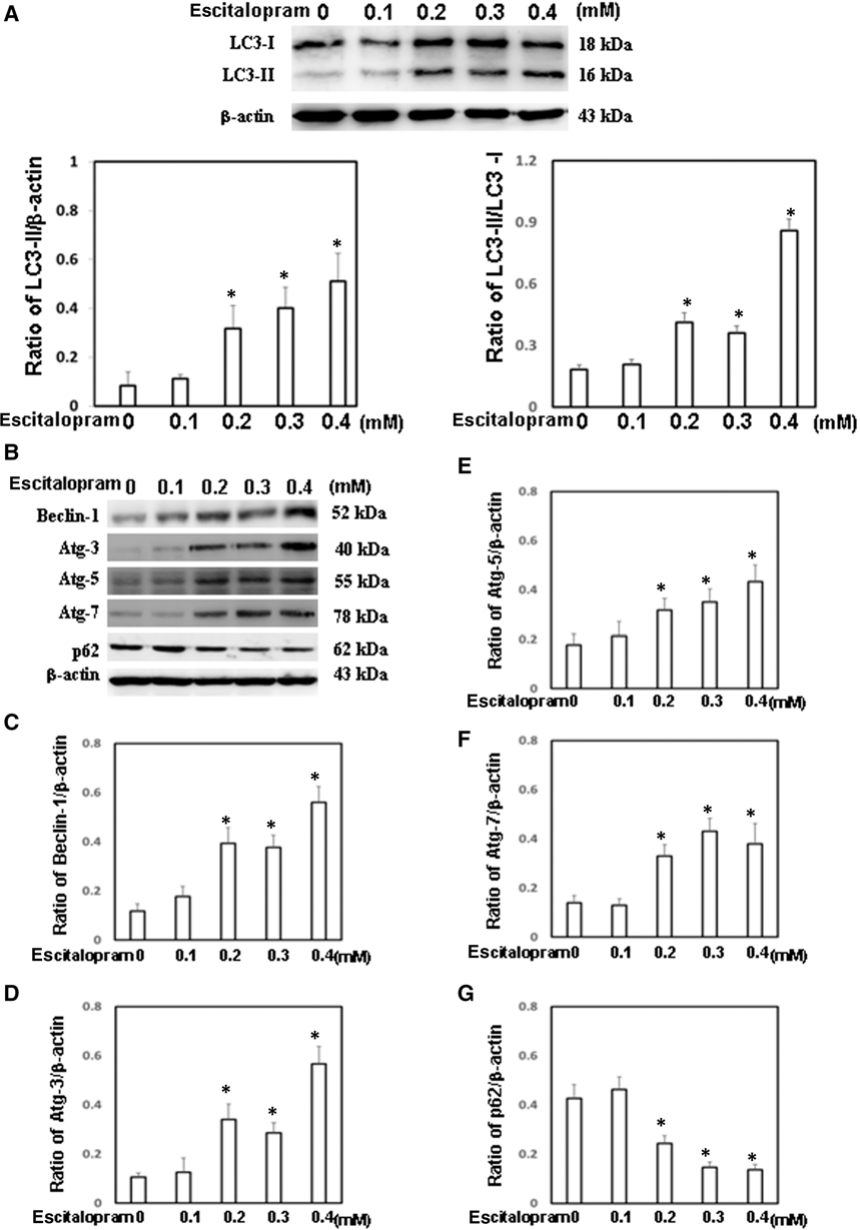
Expression of autophagy‐related proteins. (**A**) The expressions of LC3‐I and LC3‐II proteins in GBM8401 cells that were treated with different concentrations of escitalopram oxalate for 48 hrs. The ratios of LC3‐II/β‐actin and LC3‐II/LC3‐I were shown in the lower panel. (**B**) The expression of Beclin‐1, Atg‐3, Atg‐5, Atg‐7 and p62 in GBM8401 cells that were treated with different concentrations of escitalopram oxalate for 48 hrs. Bars represent the relative protein quantification of (**C**) Beclin‐1, (**D**) Atg‐3, (**E**) Atg‐5, (**F**) Atg‐7 and (**G**) p62 on the basis of β‐actin. Similar results were observed in three repeated experiments, and * indicates the significant difference, *P* < 0.05.

Although no significant cell death was observed in GBM8401 cells that were treated with different concentrations of escitalopram oxalate for 24 hrs, we further evaluate the effects of escitalopram oxalate on GBM8401 cells in the presence of escitalopram oxalate for 48 hrs. Significantly lower cell viability was detected in GBM8401 cells that were treated with 0.2, 0.3 and 0.4 mM escitalopram oxalate for 48 hrs (Fig. 5A). Significantly lower survival ratio was detected in GBM8401 cells that were treated with escitalopram oxalate for 48 hrs as compared to HBA cells (Fig. 5B). Notably, apparently higher expression of LC3‐II was observed in GBM8401 cells that were treated with 0.2, 0.3 and 0.4 mM escitalopram oxalate for 48 hrs (Fig. 5C) along with the significantly higher ratio of LC3‐II/LC3‐I (Fig. 6A). Moreover, significantly higher expressions of autophagic proteins, including Beclin‐1, Atg‐3, Atg‐5 and Atg‐7, were detected in GBM8401 cells that were treated with 0.2, 0.3 and 0.4 mM escitalopram oxalate for 48 hrs, whereas the level of p62 protein in GBM8401 significantly declined (Fig. 6B). Figures 6C–G present the quantitative results of Beclin‐1, Atg‐3, Atg‐5, Atg‐7 and p62 relative to that of β‐actin.

### Escitalopram oxalate suppresses the growth of U‐87MG cells *in vivo*

**Figure 7:**
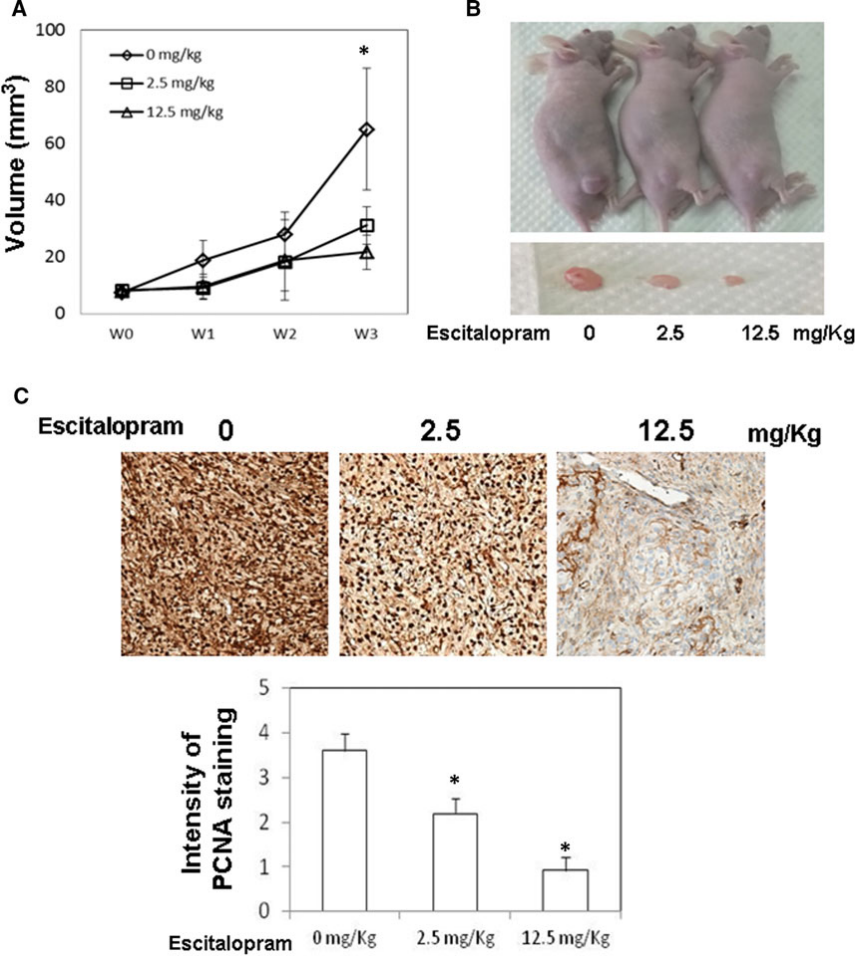
Effects of escitalopram oxalate on the growth of U‐87MG cells tumour xenograft. After injection of U‐87MG cells into the flanks of BALB/c nude mice for 1 week, the different groups of mice were daily supplemented with PBS, 2.5 mg/kg and 12.5 mg/kg escitalopram oxalate by oral gavage with for 21 days, respectively. (**A**) The volume of tumours was weekly gauged with a calliper. (**B**) Representative images of the excised xenograft tumours from different groups of mice. (**C**) Immunohistochemical stating of tumour sections from different groups of mice with an anti‐proliferating cell nuclear antigen (PCNA) antibody (at ×200 magnification). Lower panel indicates the intensity of PCNA staining, and * indicates the significant difference, *P* < 0.05.

To determine the effects of escitalopram oxalate *in vivo*, U‐87MG tumour xenografts were produced by subcutaneously injecting 5 × 10^6^ U‐87MG cells into BALB/c nude mice. When the tumour volume reached nearly 20 mm^3^, the mice were orally gavaged with PBS, 2.5 mg/kg or 12.5 mg/kg escitalopram oxalate once a day. The mean tumour volume in mice that were administered 12.5 mg/kg escitalopram oxalate was significantly smaller than those in mice that were treated with PBS or 2.5 mg/kg escitalopram oxalate (Fig. 7A). Figure 7B shows the representative xenograft tumours retrieved from the end‐point of the experiments. To examine the effect of escitalopram oxalate on xenograft U‐87MG tumour growth, immunohistochemical (IHC) staining was adopted to analyse the tumour sections by using antibodies against antiproliferating cell nuclear antigen (PCNA) antibody. Significantly reduced expression of PCNA was observed in the tumour sections from mice that were treated with 12.5 mg/kg escitalopram oxalate relative to those that were treated with PBS or 2.5 mg/kg escitalopram oxalate (Fig. 7C). The lower panel in Figure 7C presents the intensities of the tumour sections from the various groups.

## Discussion

Population‐based cohort studies have shown that neuroleptic medications are associated with a reduced cancer risk 17. However, little is known about the mechanism by which SSRI results in the death of glioma cells. This study demonstrates that escitalopram oxalate, a highly effective selective serotonin reuptake inhibitor (SSRI), significantly inhibited the proliferation and invasive ability of U‐87MG cells and significantly increased the expressions of cell cycle inhibitors. Additionally, escitalopram oxalate also induced apoptosis in U‐87MG cells and autophagy in GBM8401 cells. The *in vivo* study revealed that escitalopram oxalate suppressed the growth of xenografted U‐87MG in BALB/c nude mice. These findings are the first to suggest that escitalopram oxalate may have therapeutic potential against glioblastomas owing its antiproliferative, apoptosis‐inducible and autophagy‐inducible properties.

Cell migration is an intricate mechanism that is required for many physiological, activities such as embryogenesis, wound healing and tissue repair. However, mistakes in cell migration result in severe pathological circumstance, including cancer invasion and metastasis 18, 19. The notable features of malignant tumour cells are metastasis and invasion into surrounding tissues 20. Cancer metastatic process has four major critical steps, including detachment, migration, invasion and adhesion 21. Once the cancer cells migrate beyond the initial site, they are usually incurable and lethal 22. Actually, evidences have reported that 90% of cancer mortality is caused by cancer cell metastasis 23. Therefore, the prevention and inhibition of cancer metastasis are critical in cancer therapy. This study revealed that escitalopram oxalate significantly reduces the motility and invasive ability of U‐87MG cells, indicating its potential in inhibiting U‐87MG migration.

Increasing studies are focusing on the role of cell cycle regulatory factors such as cyclin on carcinogenesis 24. As cell cycle arrest is one of the major signals that inhibit cell proliferation 25, therefore, compounds that inhibit tumour growth by inducing cell cycle arrest and apoptosis are desired for cancer therapy 26, 27. Skp2, known as E3 ubiquitin ligase, attaches ubiquitin to its target proteins and marks them for destruction by the 26S proteasome. Skp2 is recognized as an oncogenic molecule, which targets cell cycle control elements such as p27 and p21. Increased expression of Skp2 is commonly associated with the down‐regulation of p27, p21 and p57 28. Evidences have shown that Skp2 suppression may be an excellent means of inhibiting tumorigenesis 29. Notably, escitalopram oxalate significantly inhibited Skp2 expression in U‐87MG cells, increased the expressions of cell cycle inhibitors, including P57, P21 and p27, and reduced cell viability. These experimental results revealed the cell cycle inhibitory effects of escitalopram oxalate on U‐87MG growth.

Apoptosis induction is one of the main mechanisms that impede cancer growth and is used to screen new chemotherapeutic agents against cancer 30. A possible mechanism of apoptosis is the mitochondrial apoptotic signalling pathway, which promotes the release of cytochrome c and induces apoptosis by activating caspase‐3 and PARP. PARP can be activated in cells that suffer from stress and/or DNA damage. Activated PARP can deplete the ATP of a cell, causing lysis and cell necrosis. PARP can also induce programmed cell death by producing poly‐ADP‐ribose [PAR] through stimulating mitochondria to release apoptosis‐inducing factor (AIF) 31. Herein, PARP promoted the inhibition of Bcl‐2 and triggered the release of Bax and cytochrome C (Cyt‐C), promoting apoptosis 32, 33. In this study, cytotoxic activity of escitalopram oxalate was initiated by activating caspase‐8 and cleaving caspase‐3, resulting in the proteolytic cleavage of PARP in U‐87MG cells. These findings revealed the therapeutic efficacy of escitalopram oxalate against GBM (U‐87MG) by inducing apoptosis.

Autophagy is a highly efficient style of cell death induction by excessive self‐degradation. Mounting evidences have indicated that non‐apoptotic forms of programmed cell death, especially macro‐autophagy, are novel approaches in anticancer therapy 34, 35, 36. Beclin‐1, known as an essential component of the class III PI3K complex, involves in the initiation of autophagosome formation. Atg‐3 and Atg‐7 participate in the conversion of soluble LC3‐I to lipid bound LC3‐II, which is recruited to autophagosomal membranes. Atg‐5 forms a conjugate with Atg‐12 to play a key role in autophagosome formation. The LC3‐II to LC3‐I ratio was reported to be proportional to the number of autophagic vacuoles 37. P62 contains an LC3‐interacting motif and an ubiquitin‐binding domain, which is incorporated into and degraded in autolysosomes 38. Although no apoptosis was detected in GMB8401 cells in the presence of escitalopram oxalate for 24 or 48 hrs, the current study revealed significantly increased ratio of LC3‐II/LC3‐I and elevated protein levels of Beclin‐1, Atg‐3, Atg‐5 and Atg‐7 in GBM8401 cells that were treated with 0.2, 0.3 and 0.4 mM escitalopram oxalate for 48 hrs, whereas the p62 protein levels was significantly declined. These findings demonstrated the effects of escitalopram oxalate on inducing autophagy in GBM801 cells, which also indicates a diverse mechanism of escitalopram oxalate on inhibiting different brain tumour cell lines.

The inhibitors of various pathways such as EGFR signalling, RAS/MAPK signalling and mTOR‐PI3K‐Akt‐PTEN signalling have been considered one of many future directions for GBM treatment 39. However, chemotherapy for brain cancers is commonly restricted by the poor efficacy of drug delivery through the blood–brain barrier (BBB). Indeed, almost all molecule tested cannot pass through the BBB 40, 41, 42. Although very few drug molecules can pass through the BBB; however, they cannot efficiently tell the difference between healthy and cancerous cells, leading to serious side effects in the brain 43. Therefore, imperative investigations are required to develop the drugs that can traverse the BBB and selectively kill brain tumour cells.

Recently studies have demonstrated that certain SSRIs can reduce cancer risks; however, the side effects of these SSRIs are disturbing 44. Based on decades of clinical use, escitalopram is the most selective SSRI with almost no significant affinity to other tested receptors 12. Indeed, it has been demonstrated that escitalopram has parallel therapeutic potential as citalopram and has statistically superior and clinically relevant properties compared with citalopram 45. Escitalopram reveals generally better tolerated, relatively fast onset of action, and less adverse effects as compared to most widely prescribed antidepressants 9, 10, 11, 12, 45. According to a study of clinical pharmacokinetics of escitalopram oxalate, the relevant interactions of escitalopram oxalate with other drugs are minimal because of its very poor inhibitory effects on CYP enzymes or P‐glycoprotein 11.

Evidence also indicated that the low propensity of escitalopram for cytochrome P450 (CYP)‐mediated drug–drug interactions is a potential advantage for the average adult patient, who will most likely take more than one medication in the course of long‐term therapy 11. Overall, as an allosteric serotonin reuptake inhibitor that is somewhat different from classical SSRIs, escitalopram has potential advantage in GBM therapy combined with oncology drugs.

Also known as second‐generation SSRIs for depression therapy, fluoxetine is recently reported to have selectively toxic on gliomas by generating an overloading of mitochondrial calcium ions (Ca^2+^) 8. However, evidences revealed that fluoxetine has remarkably higher adverse effects compared with escitalopram 46, 47, 48. Notably, the current study also demonstrated the cytotoxic effects on U‐87MG cells by inhibition the cell proliferation and inducing the apoptosis. Based on these superior properties than fluoxetine, escitalopram is recommended on both depression and GBM therapy. The concept of alternative mechanisms of targeting glioma that do not rely on traditional chemotherapeutic mechanisms of action is extremely important, and further investigations are required to verify alternative anti‐cancer mechanisms that can be utilized clinically to improve patient outcomes.

Finally, some limitations of the present study must be noted. First, the dosage of escitalopram oxalate used in this study was much higher than the recommended. It may raise the concern about the adverse effects of escitalopram oxalate in clinics. However, there may some solutions. Notably, there have been reports of escitalopram oxalate overdose involving doses of up to 600 mg, which is about 0.3 mM adopted in this study. According to the report by LEXAPRO (Forest Pharmaceuticals, Inc. St. Louis, MO, USA), all patients administrated with 600 mg escitalopram oxalate per day recovered and no symptoms associated with the overdoses were observed. It implies the safety and possibility of very high dose of escitalopram oxalate on administration of patients with GBM. However, lower dose of escitalopram oxalate will be suggested in further evaluation on treatment with GBM as 0.1 ~ 0.2 mM escitalopram oxalate also significantly inhibited the proliferation and invasive ability on U‐87MG. Second, escitalopram oxalate is recommend for certain psychological disorders such as major depressive disorder (MDD) and generalized anxiety disorder (GAD) but not GBM. Therefore, combination of escitalopram oxalate with certain GBM medicines such as temozolomide (TMZ) or bevacizumab (BZV) on GBM are required to further evaluate their effects in clinics. Third, we only subcutaneously injected U‐87MG cells into the flank of the mice to evaluate the effects of escitalopram oxalate. It cannot recapitulate key features of human tumours. Therefore, a preclinical orthotopic model with intracranial injection of GBM cells will be adopted for further identifying the mechanism of escitalopram oxalate on GBM. However, with these caveats accepted, the results presented in this study imply a possibility that escitalopram oxalate may have therapeutic potential on administration of patients with GBM.

## Conclusions

Although GBM remains one of the most arduous cancer types, this study provides experimental evidence that escitalopram oxalate is a potent therapeutic drug against GBM probably owing to its ability to pass through the BBB and have direct pharmaceutical effects in the brain. However, further preclinical orthotopic model will be adopted for identifying the precise mechanism of Escitalopram oxalate on glioblastoma.

## Author contributions

V.C.C., T.C.H., B.S.T. conceived and designed the experiments. Y.H.H., L.J.C. performed the experiments. T.C.H., L.J.C., B.S.T. analyzed the data. V.C.C., Y.H.H., B.S.T., T.C.H. contributed reagents/materials/analysis tools. B.S.T., T.C.H. wrote the manuscript. All authors have read and approved the final version of this manuscript.

## Conflict of interest

No conflict of interest.
